# Plasma Phospho‐Tau Identifies Alzheimer's Co‐Pathology in Patients with Lewy Body Disease

**DOI:** 10.1002/mds.28370

**Published:** 2020-12-07

**Authors:** Sara Hall, Shorena Janelidze, Elisabet Londos, Antoine Leuzy, Erik Stomrud, Jeffrey L. Dage, Oskar Hansson

**Affiliations:** ^1^ Clinical Memory Research Unit, Department of Clinical Sciences Malmö Lund University Lund Sweden; ^2^ Memory Clinic Skåne University Hospital Malmö Sweden; ^3^ Eli Lilly and Company Indianapolis IN USA

**Keywords:** plasma, phospho‐tau, Parkinson's disease dementia, dementia with Lewy bodies, biomarkers

## Abstract

**Background:**

Alzheimer's disease co‐pathology is common in dementia with Lewy bodies and Parkinson's disease with dementia (Lewy body disease) and can reliably be detected with positron emission tomography (PET) or cerebrospinal fluid (CSF) biomarkers. Recently developed blood biomarkers are more accessible and less expensive alternatives.

**Objective:**

To investigate if plasma phospho‐tau217 and phospho‐tau181 can detect Alzheimer's pathology in Lewy body disease with dementia.

**Methods:**

In this cross‐sectional study we investigated plasma phospho‐tau217 and phospho‐tau181 in 35 patients with Lewy body disease with dementia. Patients underwent tau‐PET imaging (^18^F‐RO948).

**Results:**

Plasma phospho‐tau217 correlated with plasma phospho‐tau181, CSF phospho‐tau217 (r_s_ = 0.68, *P* < 0.001), and negatively with CSF β‐amyloid_42/40_ (r_s_ = −0.52, *P* = 0.001). Plasma phospho‐tau217 and phospho‐tau181 correlated with tau‐PET signal in the temporal cortex (r_s_ > 0.56, *P* < 0.001) and predicted abnormal tau‐PET status and β‐amyloid status (area under the curve > 0.78 and > 0.81, respectively).

**Conclusion:**

Plasma phospho‐tau might be a useful marker for Alzheimer's co‐pathology in Lewy body disease with dementia. © 2020 The Authors. *Movement Disorders* published by Wiley Periodicals LLC on behalf of International Parkinson and Movement Disorder Society.

## 1 Introduction

Dementia with Lewy bodies (DLB) and Parkinson's disease with dementia (PDD) are major neurocognitive disorders characterized by subcortical and cortical intracellular inclusions (Lewy bodies and Lewy neurites) containing α‐synuclein (α‐syn).^1^, ^2^ Although there are clinical, neuropathological, and genetic differences between DLB and PDD, the overlap is substantial.^3^ In DLB and PDD, spread and increased burden of α‐syn pathology in cortical and limbic regions correlate with cognitive decline.^4^, ^5^, ^6^ Concomitant Alzheimer's disease (AD) is common at autopsy in DLB, but also in PDD^7^, ^8^ and AD‐related tau and β‐amyloid (Aβ) pathologies have been shown to contribute independently to dementia.^4^, ^9^ This suggests that it will be important to identify the pathological substrates of cognitive decline when selecting patients with DLB or PDD for future clinical trials of drugs targeting specific pathologies. AD co‐pathology in DLB or PDD can be detected using cerebrospinal fluid (CSF) and positron emission tomography (PET) biomarkers. Cognitive impairment in DLB and PDD have been associated with CSF AD biomarkers (tau phosphorylated at threonine 181 [P‐tau181], total tau and Aβ_42_),^10^ and tau‐PET measures.^11^, ^12^ However, limited availability, cost, and invasiveness hamper global implementation in identifying AD pathology in clinical settings and clinical trials. Blood P‐tau181 is a promising diagnostic and prognostic biomarker of AD, correlating strongly with CSF P‐tau181, and accurately identifying individuals with abnormal tau‐PET and Aβ‐PET.^13^, ^14^ While assays detecting P‐tau181 in CSF have dominated AD biomarker research,^15^ new studies comparing P‐tau181 with tau phosphorylated at threonine 217 (P‐tau217) in both CSF and blood have shown that P‐tau217 better predict AD‐related Aβ and tau pathologies.^16^, ^17^, ^18^ Here we investigated whether plasma P‐tau217 and plasma P‐tau181 could be used as biomarkers of AD co‐pathology in patients with DLB or PDD. We assessed the accuracy of plasma P‐tau217 and plasma P‐tau181 (and for comparison CSF P‐tau217 and CSF P‐tau181) to predict tau‐PET and CSF Aβ positivity in DLB or PDD.

## 2 Methods

### 2.1 Study Participants

A total of 35 patients with DLB (n = 30) or PDD (n = 5) were included between 2017 and 2019, all part of the Swedish BioFINDER‐2 study.^19^ In this convenience cohort, all available plasma samples from patients with DLB or PDD were analyzed. Patients met criteria for DLB or PDD according to *Diagnostic and Statistical Manual of Mental Disorders* (DSM‐5).^20^ Participants underwent a medical history, neurologic examination, cognitive testing, lumbar puncture, blood sampling, and tau‐PET imaging using [^18^F]RO948.^21^ Cognitive battery is described in Appendix S1. The study procedure was approved by the local ethics committee at Lund University Sweden and conducted according to the Declaration of Helsinki. All study participants gave written informed consent.

### 2.2 CSF and Plasma Sampling and Analysis

CSF samples were obtained by lumbar puncture in the morning, with collection and analyses performed according to a standardized protocol. Plasma samples were obtained at the time of lumbar puncture. CSF and plasma analyses are described in Appendix S1. Aβ positivity was defined as CSF Aβ_42_/Aβ_40_ ratio < 0.752, determined using mixture modeling.^18^

### 2.3 Tau‐PET Imaging and Processing

Tau‐PET images were acquired using digital GE Discovery MI scanners 70–90 minutes post injection of ~370 MBq [^18^F]RO948^21^ as described in Appendix S1.^19^

### 2.4 Statistical Analysis

Mann–Whitney U test was used for comparison of continuous variables between groups and chi‐square test for dichotomous variables. Univariate associations between two continuous variables were analyzed using Spearman ρ. To investigate the performance of biomarkers in predicting tau‐PET positivity and CSF Aβ positivity receiver operating characteristic (ROC) analysis was performed. For further information on statistical analysis see Appendix S1.

## 3 Results

### 3.1 Demographics

**FIG. 1.**
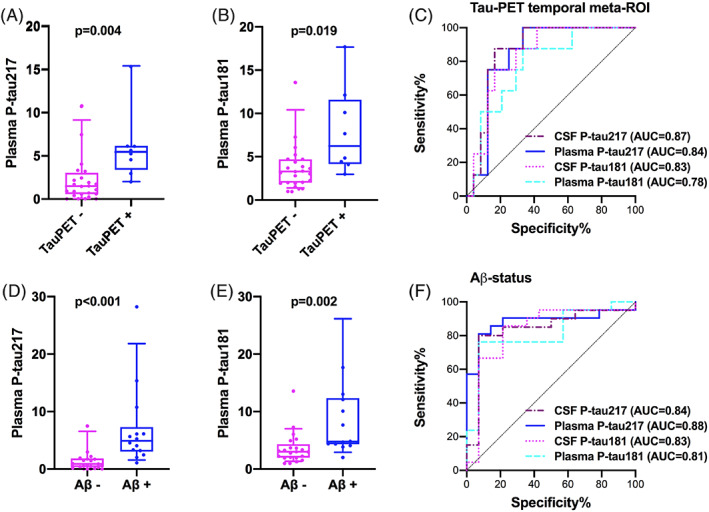
Plasma P‐tau levels according to Tau‐positron emission tomography (PET) (**A,B**) and cerebrospinal fluid (CSF)‐Aβ (**D,E**) status. Receiver operating characteristic (ROC) curves of plasma and CSF P‐tau for Tau‐PET (**C**) and CSF‐Aβ (**F**) status. Two outliers are not shown in the figure but were included in the statistical analysis. AUC, area under the curve.

The study included 35 DLB/PDD patients with mean age 73.5 (SD 6.5) years; six were females. Participants were stratified according to tau‐PET status in the temporal meta‐region of interest (ROI) where abnormal binding of tau‐PET tracers is typical in AD^22^, ^23^ but has also been reported in DLB/PDD.^12^ Demographic and clinical characteristics as well as plasma and CSF biomarker concentrations are presented in Table 1. There were no significant differences in age or sex between DLB/PDD patients with normal and abnormal tau‐PET. Plasma P‐tau217 and P‐tau181 did not differ by age or sex. Patients with elevated tau‐PET standardized uptake value ratio (SUVR) had higher levels of plasma P‐tau217 (*P* = 0.004) and plasma P‐tau181 (*P* = 0.019) compared to those with normal tau‐PET (Fig. 1A,B). Similar findings were seen in CSF P‐tau217 and P‐tau181 (*P* = 0.002 and *P* = 0.005) (Table 1).

**TABLE 1:** Participant characteristics

Characteristic	DLB/PDD with normal tau‐PET (n = 24)	DLB/PDD with abnormal tau‐PET (n = 8)	*P* value	All DLB/PDD (n = 35)
Sex female/male (% females)	5/19 (21%)	1/7 (13%)	NS	6/29
Age (yr)	73.4 (6.1)	72.6 (8.5)	NS	73.5 (6.5)
Education (yr)	11.3 (3.4)	15.5 (3.9)	0.009	12.2. (4.1)
MMSE	22.5 (4.8) (1 missing)	21.9 (6.6)	NS	22.4 (5.0)
Word‐list delayed recall (ADAS‐Cog)	7.1 (2.1) (3 missing)	7.7 (2.0) (2 missing)	NS	7.2 (2.0)
Animal fluency	11.9 (4.2) (5 missing)	19.2 (5.9) (2 missing)	0.006	11.5 (5.3)
Letter S fluency	5.5 (3.1) (8 missing)	11.8 (6.4) (2 missing)	0.012	6.8 (4.8)
Aβ positivity	6 (25%)	7 (86%)	0.002	14 (40%)
CSF Aβ_42/40_	1.01 (0.70–1.23)	0.61 (0.56–0.67)	0.004	0.88 (0.59–1.16)
*APOE* ε4 positivity	7 (29%) (1 missing)	6 (75%)	0.028	15/19 (44%)
Plasma P‐tau217 (pg/mL)	1.49 (0.58–3.28)	5.46 (3.37–6.14)	0.004	1.92 (0.61–5.26)
Plasma P‐tau181 (pg/mL)	3.29 (2.01–4.71)	6.25 (4.18–11.60)	0.019	3.84 (2.24–6.07)
CSF P‐tau217 (pg/mL)	60.5 (38.8–146.7)	210.8 (176.8–300.0)	0.002	97.1 (49.4–195.7)
CSF P‐tau181 (pg/mL)	49.8. (36.4–84.7)	103.3 (78.2–128.9)	0.005	58.9 (39.6–99.1)

Demographic data are given as mean and standard deviation, except dichotomous values. Due to outliers, biomarkers are given as median and interquartile range. Group differences were analyzed using the Mann–Whitney test. For sex, Aβ‐status, and *APOE* ε4 positivity, the chi‐square test was used. Valid imaging data were missing for three cases.

DLB, dementia with Lewy bodies; PDD, Parkinson's disease with dementia; PET, positron emission tomography; MMSE, Mini‐Mental State Examination; ADAS‐Cog, Alzheimer's Disease Assessment Scale‐Cognitive Subscale; APOE, apolipoprotein E; CSF, cerebrospinal fluid.

### 3.2 Correlations between Plasma and CSF P‐Tau Biomarkers

Correlations between the biomarkers are given in Appendix S1, Table S1. Plasma P‐tau217 correlated with plasma P‐tau181 (r_s_ = 0.68, *P* < 0.001) and with CSF P‐tau217 (r_s_ = 0.68, *P* < 0.001). Plasma P‐tau181 correlated with CSF P‐tau181 (r_s_ = 0.55, *P* < 0.001). Higher levels of plasma P‐tau217 and P‐tau 181 were associated with lower CSF Aβ_42_/Aβ_40_ ratio (r_s_ = −0.52, *P* = 0.001 and r_s_ = −0.50, *P* = 0.002). There were no significant correlations between plasma and CSF P‐tau217 and P‐tau181, respectively, when investigating the correlations in Aβ‐positive and Aβ‐negative individuals separately.

### 3.3 Correlations between Plasma P‐Tau and Tau‐PET

Increased plasma P‐tau217 and P‐tau181 concentrations were associated with higher uptake on tau‐PET SUVR in the temporal meta‐ROI (r_s_ = 0.57, *P* < 0.001 and r_s_ = 0.66, *P* < 0.001). These results were comparable to the associations between tau‐PET and CSF P‐tau (CSF P‐tau217: r_s_ = 0.65, *P* < 0.001; CSF P‐tau181: r_s_ = 0.59, *P* = 0.002). When investigating these correlations in Aβ‐positive and Aβ‐negative individuals separately, a significant correlation was seen between plasma P‐tau217 and tau‐PET in Aβ‐positive participants (r_s_ = 0.57, *P* = 0.044). Using voxelwise multiple regression, higher levels of plasma P‐tau217 and to a lesser extent P‐tau181 were associated with higher tau‐PET SUVR, predominantly in the lateral temporal lobes (*P* < 0.001, k ≥ 40) (Figure S1 in Appendix S1).

### 3.4 Prediction of Tau‐PET Outcome Using Plasma P‐Tau

Plasma P‐tau217 predicted tau‐PET positivity in the temporal meta‐ROI with an area under the curve (AUC) of 0.84 (95% CI 0.71–0.98). The corresponding AUC for plasma P‐tau181 was 0.78 (95% CI 0.61–0.95). The performance of plasma P‐tau217 was similar to CSF P‐tau217 (AUC 0.87, 95% CI 0.74–0.99) and CSF P‐tau181 (AUC 0.83, 95% CI 0.69–0.97) (Fig. 1C).

### 3.5 Prediction of Aβ‐Status Using Plasma P‐Tau

Aβ‐positive subjects with DLB/PDD had higher levels of P‐tau217 and P‐tau181 in plasma compared to Aβ‐negative individuals (*P* < 0.001 and *P* = 0.002) (Fig. 1D,E). Similar findings were seen in CSF P‐tau217 and P‐tau181 (*P* < 0.001 and *P* = 0.001). Furthermore, plasma P‐tau217 and P‐tau181 predicted Aβ‐status (AUC 0.88, 95% CI 0.76–1.00 and AUC 0.81, 95% CI 0.66–0.96, respectively) with similar accuracy to CSF P‐tau217 (AUC 0.84, 95% CI 0.69–0.99) and CSF P‐tau181 (AUC 0.83, 95% CI 0.67–0.99) (Fig. 1F).

### 3.6 Sensitivity Analyses

Two outliers were identified with plasma P‐Tau217 and P‐Tau181 values 3 standard deviations (SD) above the mean. Excluding these individuals did not change the overall results (Tables S2, S3, and Figure S2). Seven participants with plasma P‐tau217 levels below the detection limit of the assay (0.48 pg/mL) were included in the main analysis. Results of the sensitivity analysis excluding values below the detection limit were similar to the main results (Appendix S1).

## 4 Discussion

In the present study of patients with DLB or PDD, we find correlations between plasma and CSF levels of P‐tau217 and P‐tau181. Higher plasma levels of P‐tau217 and P‐tau181 were also associated with lower CSF Aβ_42_/Aβ_40_ and with higher tau‐PET SUVR. Furthermore, plasma P‐tau217 and P‐tau181 identified AD co‐pathology in patients with DLB or PDD by predicting both tau‐PET positivity and CSF Aβ positivity with a relatively high accuracy (AUC 0.78–0.88).

Associations between CSF and plasma levels of both P‐tau217 and P‐tau181 have been reported, indicating that increased phosphorylation of tau in the AD brain leads to changes in P‐tau levels in blood as well as in CSF.^13^, ^14^, ^18^ In line with these data, we observed significant correlations between plasma and CSF P‐tau biomarkers in DLB and PDD patients. Further, in the present study we found significant correlations between plasma P‐tau and tau‐PET SUVR suggesting that in individuals with DLB or PDD, AD‐related changes in tau metabolism are reflected in the blood levels of P‐tau. The results also indicated that plasma P‐tau217 and P‐tau181 identified DLB or PDD with abnormal tau‐PET SUVR in the temporal meta‐ROI (AUC 0.84 and 0.78, respectively).

The previously reported correlations between tau PET and plasma P‐tau217 or P‐tau181 were significant in Aβ‐positive, but not Aβ‐negative, individuals.^13^, ^14^, ^18^ Here, plasma P‐tau217 correlated with tau‐PET even when restricting the analysis to the Aβ‐positive cases, which was not the case for P‐tau181, where a significant association was only seen in the whole cohort, likely due to the small number of Aβ‐positive cases. As expected no correlations were seen in Aβ‐negative cases.

Autopsy and PET studies have shown that abnormal intracerebral Aβ accumulation might be common in DLB and PDD.^8^, ^24^ The ratio of CSF Aβ_42_ to Aβ_40_ is a well‐established biomarker of increased brain amyloid deposition.^25^ Here we report that plasma P‐tau217 and P‐tau181 correlated with CSF Aβ_42_/Aβ_40_ and could accurately detect CSF Aβ positivity (AUC 0.88 and 0.81, respectively) in DLB or PDD.

In DLB and PDD, Aβ and tau pathologies may act synergistically with α‐syn pathology influencing the clinical presentation and prognosis.^3^ Indeed, a recent study on autopsy‐confirmed DLB found that phenotype and dementia trajectory were associated with the distribution of both α‐syn and tau pathology.^26^ Effects of new disease‐modifying treatments will most likely differ across patient populations with single and mixed pathologies. Consequently, identification of concomitant AD in patients with DLB or Parkinson's disease might be of critical importance for patient selection and stratification in future clinical trials. Although, CSF and PET imaging biomarkers hold promise, in this study we show, to the best of our knowledge for the first time, that plasma P‐tau might be an accurate marker of AD co‐pathology in DLB and PDD.

In the present study, participants are consecutively included patients referred to a memory clinic where they are investigated by an experienced physician to ascertain as certain a diagnosis as possible. However, these results need to be validated in a primary care setting. Limitations are the very small sample size, the cross‐sectional study design, and lack of neuropathology. Although previous reports suggested that P‐tau217 might outperform P‐tau181 as a biomarker of AD,^18^ the present study was underpowered to detect statistically significant differences in biomarker performance. Longitudinal analyses in larger cohorts are needed to further investigate the utility of plasma P‐tau217 and P‐tau181 in the prognosis of cognitive decline in patients with Lewy body disease. Finally, the plasma P‐tau assays used in the present study are research‐grade, and a clinical‐grade assay needs to be developed for it to be a clinically useful biomarker.

In conclusion, we show that plasma P‐tau might identify tau and Aβ pathologies in DLB or PDD with a high diagnostic accuracy. Although, plasma P‐tau may prove to have significant utility in detection of concomitant AD in patients with DLB or PDD, further studies are needed to substantiate its performance and validate its use prior to implementation in a clinical setting or in clinical trials.

## Author Roles

(1) Research Project: A. Conception, B. Organization, C. Execution; (2) Statistical Analysis: A. Design, B. Execution, C. Review and Critique; (3) Manuscript: A. Writing of the First Draft, B. Review and Critique.

S.H.: 1C, 2A, 2B, 3A

S.J.: 1C, 2B, 2C, 3A, 3B

E.L.: 1B, 2C, 3B

A.L: 1B, 2B, 2C, 3B

E.S.: 1B, 2C, 3B

J.L.D.: 1C, 2C, 3B

O.H.: 1A, 1B, 2A, 2C, 3B

## Data Access and Responsibility

O.H. and S.H. take responsibility for the integrity of the data and the accuracy of the data analysis.

## Full Financial Disclosure for the Previous 12 Months

S.H. has received research support from the Greta and Johan Kock Foundation (paid to the institution).

S.J., E.L., A.L., and E.S. have nothing to disclose.

J.L.D. is an employee and stock holder of Eli Lilly and Company.

O.H. has acquired research support (for the institution) from Roche, Pfizer, GE Healthcare, Biogen, Eli Lilly, and AVID Radiopharmaceuticals. In the past 12 months he has received consultancy/speaker fees (paid to the institution) from Biogen and Roche.

## Funding Sources for Study

Work at the authors' research centre was supported by the Swedish Research Council, the Knut and Alice Wallenberg Foundation, the Marianne and Marcus Wallenberg Foundation, the Strategic Research Area MultiPark (Multidisciplinary Research in Parkinson's disease) at Lund University, the Swedish Alzheimer Foundation, the Swedish Brain Foundation, The Parkinson Foundation of Sweden, The Parkinson Research Foundation, the Skåne University Hospital Foundation, and the Swedish federal government under the ALF agreement. The precursor of ^18^F‐RO948 was provided by Roche.

## Supporting information


**Appendix S1**. Supporting Information.
