# Supplementary material for: Plasma Phospho‐Tau Identifies Alzheimer's Co‐Pathology in Patients with Lewy Body Disease
Source: Mov Disord. 2020 Dec 7;36(3):767–71. doi: 10.1002/mds.28370 (PMC8048822; doi:10.1002/mds.28370)
Supplement: Supplementary file 1 — Appendix S1. Supporting Information. [file MDS-36-767-s001.docx]

**Supporting Information**

**Plasma phospho-tau identifies Alzheimer’s co-pathology in patients with Lewy body disease**

Sara Hall, Shorena Janelidze, Elisabet Londos, Antoine Leuzy, Erik Stomrud, Jeffrey L. Dage, and Oskar Hansson

**Cognitive tests**

The study participants’ cognitive function was assessed using e.g. the Mini Mental State Examination (MMSE), 10 word-list immediate and delayed recall test of the Alzheimer’s Disease Assessment Scale (ADAS), the one-minute phonetic verbal fluency test (Letter S Fluency test) and one-minute animal fluency test.^1-4^

**CSF and Plasma analysis**

CSF concentrations of plasma and CSF P-tau217 were measured using Mesoscale Discovery (MSD) based immunoassays at Lilly Research Laboratories, IN, USA by scientists blinded to the clinical and imaging data. For plasma analysis, biotinylated‐IBA493 was used as a capture antibody and SULFO‐TAG‐4G10‐E2 (anti‐Tau) as the detector. For CSF analysis, biotinylated‐IBA413 was used as a capture antibody and a tau‐specific antibody (LRL) as the detector. Plasma and CSF samples were diluted 1:2 and 1:8, respectively, in sample buffer containing heterophilic blocking reagent 1 at a concentration of 200 μg/ml (Scantibodies Inc). Both plasma and CSF assays were calibrated using a recombinant tau (4R2N) protein that was phosphorylated in vitro using a reaction with glycogen synthase kinase‐3 and characterized by mass spectrometry.

CSF Aβ_42_ and Aβ_40_ were measured at the Clinical Neurochemistry Laboratory in Gothenburg with Meso Scale Discovery immunoassays (MSD; Rockville, MD, USA).

CSF and plasma P-tau181 were analyzed as previously described.^5^ The procedure and analysis of CSF followed the Alzheimer’s Association Flow Chart for CSF biomarkers.^6^

**Tau-PET imaging and processing**

Tau-PET images were acquired using digital GE Discovery MI scanners 70-90 min post injection of ~370 MBq [^18^F]RO948^7^. Standardized uptake value ratio (SUVR) images were created using the inferior cerebellar cortex as reference region^8^. A volume weighted FreeSurfer-based temporal meta-region of interest (ROI) was created, including entorhinal cortex, fusiform gyrus, parahippocampal cortex, amygdala, and inferior and middle temporal cortices.^9^ Thresholds for defining tau-PET positivity within these ROIs were defined using a priori cutoff of >1.36 based on the mean SUVR within a given region plus 2.5 standard deviations (SD) in young Aβ-negative controls.^10^ Valid imaging data was missing in 3 individuals.

**Statistical Analyses**

Associations between plasma P-tau (217 and 181, log-transformed) and [^18^F]RO948 SUVR were further analyzed at the voxel level using multiple regression models including age and sex, as implemented in SPM12. Due to relatively small size, the study was underpowered to detect differences in the performance of plasma P-tau217, plasma P-tau181, CSF P-tau217 and CSF P-tau181.

P<0.05 (two-sided) was considered statistically significant. SPSS (version 26; SPSS Inc., Chicago, Illinois), R version 3.4.3 (RStudio) and GraphPad Prism version 8 were used for statistical analyses.

**Table S1. Correlations between biomarkers**

|  | Plasma P-tau181 | CSF  P-tau217 | CSF  P-tau181 | CSF Aβ_42/40_ |
| --- | --- | --- | --- | --- |
| Plasma P-tau217 | 0.68 *** | 0.68 *** | 0.63 *** | -0.52 ** |
| Plasma P-tau181 | - | 0.57 *** | 0.55 *** | -0.50 ** |
| CSF P-tau217 | - | - | 0.96 *** | -0.56 *** |
| CSF P-tau181 | - | - | - | -0.49 ** |

Correlations were calculated using Spearman Rank. Correlation coefficients are given as Spearman ρ. *** p<0.001 ** p<0.01.

**Figure S1**

**
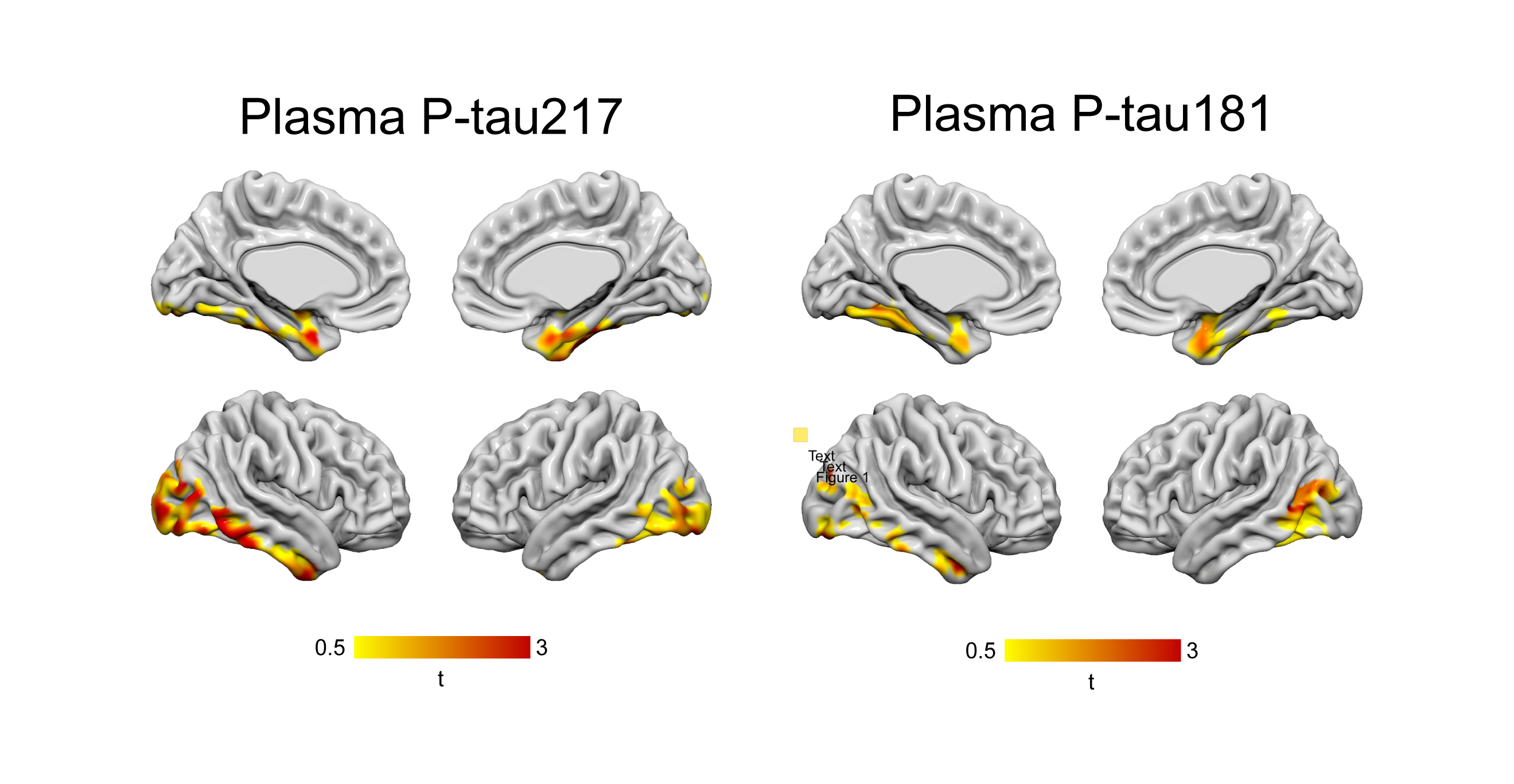
**

**Fig. S1.** Voxel-based associations between plasma P-tau217 and P-tau181 (both log-10) and [^18^F]RO948 SUVR, adjusted for age and sex (corrected for multiple testing using false discovery rate, P<0.05, k>40). Color bar shows t-values, with higher values indicating stronger associations.

**Results with outliers excluded**

Two outliers were identified with plasma P-tau217 and P-tau181 values 3 standard deviations (SD) above the mean. Excluding these individuals did not change the overall results (Table S2, Table S3 and Fig. S2)

**Table S2. Correlations between biomarkers, outliers excluded**

|  | Plasma P-tau181 | CSF  P-tau217 | CSF  P-tau181 | CSF Aβ_42/40_ |
| --- | --- | --- | --- | --- |
| Plasma P-tau217 | 0.66 *** | 0.70 *** | 0.70 *** | -0.53 ** |
| Plasma P-tau181 | - | 0.60 *** | 0.60 *** | -0.51 ** |
| CSF P-tau217 | - | - | 0.97 *** | -0.54 ** |
| CSF P-tau181 | - | - | - | -0.48 ** |

Correlation was calculated using Spearman ρ. *** p<0.001 ** p<0.01.

**Table S3. Correlations between biomarkers and uptake on tau-PET, outliers excluded**

|  | Tau-PET temporal meta-ROI |
| --- | --- |
| Plasma P-tau217 | r_s_ = 0.63, p<0.001 |
| Plasma P-tau181 | r_s_ = 0.71, p<0.001 |
|  |  |
| CSF P-tau217 | r_s_ = 0.63, p<0.001 |
| CSF P-tau181 | r_s_ = 0.58, p<0.002 |

Correlations were using calculated Spearman ρ.

**Figure S2**

**Fig. S2.** ROC curves showing the predictive performance of P-tau217 and P-tau181 in plasma and CSF on A) tau-PET in the temporal meta-ROI (positive N=8 and negative N= 22) and B) Aβ-status (positive N=13 and negative N= 20).

**Results excluding plasma P-tau217 values below the lower detection limit of the assay**

We performed sensitivity analysis excluding seven participants with plasma P-tau217 levels below the detection limit of the assay (0.48 pg/mL).

The difference in plasma P-tau217, P-tau181, CSF P-tau217 and CSF P-tau181 concentrations between individuals with abnormal tau-PET (n=8) and normal tau-PET (n=19) were very similar to the main findings of the study.

Plasma P-tau217 correlated with plasma P-tau181 (r_s_=0.74, p<0.001). Further, positive correlations were found between plasma P-tau217 and both CSF P-tau217 (r_s_=0.57, p=0.001) and CSF P-tau181 (r_s_=0.53, p=0.004). A negative correlation was seen between plasma P-tau217 and Aβ_42_/Aβ_40_ ratio (r_s_=-0.49, p=0.009).

Plasma P-tau217 correlated with uptake on tau-PET SUVR in the temporal meta-ROIs (r_s_=0.43, p=0.026). Plasma P-tau217 predicted tau-PET positivity in the temporal meta-ROI with a ROC derived AUC of 0.80 (95% CI 0.64-0.97).

Aβ-positive (n=14) subjects had higher levels of P-tau217 in plasma compared with Aβ-negative individuals (n=14) (p<0.004) and plasma P-tau217 predicted Aβ-status with an AUC of 0.82 (95% CI 0.65-1.00).

**References**

1. Folstein MF, Folstein SE, McHugh PR. "Mini-mental state". A practical method for grading the cognitive state of patients for the clinician. Journal of psychiatric research. 1975;12:189-198

2. Mohs RC, Cohen L. Alzheimer's disease assessment scale (adas). Psychopharmacology bulletin. 1988;24:627-628

3. Isella V, Mapelli C, Siri C, et al. Validation and attempts of revision of the mds-recommended tests for the screening of parkinson's disease dementia. Parkinsonism & related disorders. 2014;20:32-36

4. Sebaldt R, Dalziel W, Massoud F, et al. Detection of cognitive impairment and dementia using the animal fluency test: The decide study. Can J Neurol Sci. 2009;36:599-604

5. Janelidze S, Mattsson N, Palmqvist S, et al. Plasma p-tau181 in alzheimer's disease: Relationship to other biomarkers, differential diagnosis, neuropathology and longitudinal progression to alzheimer's dementia. Nat Med. 2020;26:379-386

6. Blennow K, Hampel H, Weiner M, Zetterberg H. Cerebrospinal fluid and plasma biomarkers in alzheimer disease. Nat Rev Neurol. 2010;6:131-144

7. Smith R, Scholl M, Leuzy A, et al. Head-to-head comparison of tau positron emission tomography tracers [(18)f]flortaucipir and [(18)f]ro948. Eur J Nucl Med Mol Imaging. 2020;47:342-354

8. Baker SL, Maass A, Jagust WJ. Considerations and code for partial volume correcting [(18)f]-av-1451 tau pet data. Data Brief. 2017;15:648-657

9. Ossenkoppele R, Rabinovici GD, Smith R, et al. Discriminative accuracy of [18f]flortaucipir positron emission tomography for alzheimer disease vs other neurodegenerative disorders. JAMA. 2018;320:1151-1162

10. Leuzy A, Smith R, Ossenkoppele R, et al. Diagnostic performance of ro948 f 18 tau positron emission tomography in the differentiation of alzheimer disease from other neurodegenerative disorders. JAMA Neurol. 2020
